# Coexistence of nitrifying, anammox and denitrifying bacteria in a sequencing batch reactor

**DOI:** 10.3389/fmicb.2014.00028

**Published:** 2014-02-04

**Authors:** Michela Langone, Jia Yan, Suzanne C. M. Haaijer, Huub J. M. Op den Camp, Mike S. M. Jetten, Gianni Andreottola

**Affiliations:** ^1^Department of Civil, Environmental and Mechanical Engineering, University of TrentoTrento, Italy; ^2^Department of Microbiology, Institute for Water and Wetland Research, Radboud University NijmegenNijmegen, Netherlands; ^3^Department of Environmental Engineering, College of Environment and Energy, South China University of TechnologyGuangzhou, China

**Keywords:** *amo*A, Brocadia, denitrifiers, n-damo, *nir*S, Nitrobacter, *pmo*A, SNAD

## Abstract

Elevated nitrogen removal efficiencies from ammonium-rich wastewaters have been demonstrated by several applications, that combine nitritation and anammox processes. Denitrification will occur simultaneously when organic carbon is also present. In this study, the activity of aerobic ammonia oxidizing, anammox and denitrifying bacteria in a full scale sequencing batch reactor, treating digester supernatants, was studied by means of batch-assays. AOB and anammox activities were maximum at pH of 8.0 and 7.8–8.0, respectively. Short term effect of nitrite on anammox activity was studied, showing nitrite up to 42 mg/L did not result in inhibition. Both denitrification via nitrate and nitrite were measured. To reduce nitrite-oxidizing activity, high NH_3_-N (1.9–10 mg NH_3_-N/L) and low nitrite (3–8 mg TNN/L) are required conditions during the whole SBR cycle. Molecular analysis showed the nitritation-anammox sludge harbored a high microbial diversity, where each microorganism has a specific role. Using ammonia monooxygenase α–subunit (*amo*A) gene as a marker, our analyses suggested different macro- and micro-environments in the reactor strongly affect the AOB community, allowing the development of different AOB species, such as *N. europaea/eutropha* and *N. oligotropha* groups, which improve the stability of nitritation process. A specific PCR primer set, used to target the 16S rRNA gene of anammox bacteria, confirmed the presence of the “*Ca*. Brocadia fulgida” type, able to grow in presence of organic matter and to tolerate high nitrite concentrations. The diversity of denitrifiers was assessed by using dissimilatory nitrite reductase (*nir*S) gene-based analyses, who showed denitifiers were related to different betaproteobacterial genera, such as *Thauera*, *Pseudomonas*, *Dechloromonas* and *Aromatoleum*, able to assist in forming microbial aggregates. Concerning possible secondary processes, no n-damo bacteria were found while NOB from the genus *Nitrobacter* was detected.

## Introduction

New nitrogen removal pathways were recently discovered and complex biological nitrogen removal processes have been developed in WWTPs, as alternatives to the traditional nitrification–denitrification process, requiring specific knowledge on microbial community composition and physiology. For instance, the nitritation process followed by denitrification via nitrite (Ruiz et al., 2006) has been proposed as a more energy and cost-effective process than conventional full nitrification-denitrification process, due to the elimination of the nitrite oxidation step and the following nitrate reduction. Further innovation came from the enrichment of the anaerobic ammonium oxidizing (anammox) bacteria (Strous et al., 1999), able to convert ammonium with nitrite under anaerobic conditions without the need for addition of electron donors, saving energy, and resource.

A challenging wastewater treatment application is the combination of nitritation with the anammox process. It has been implemented for nitrogen removal from ammonium-rich wastewaters, such as digester supernatants, in various WWTPs around the globe (Wett et al., 1998; Joss et al., 2009; Vázquez-Padín et al., 2009). Different reactor configurations, under oxygen-limited conditions, have been applied, as reported by Van Hulle et al. (2010), including both two—and one—reactor systems, using either activated sludge or biofilm or granular biomass.

Nevertheless, treating digester supernatants, a little amount of organic carbon is present in the effluents and, thus, denitrifiers could proliferate together with nitrifiers and anammox bacteria, improving or compromising the nitrogen removal efficiency.

The coexistence of denitrifiers and nitrifiers has been found of importance in wastewater treatment plants for nitrogen removal and it is well studied. On the contrary, only recently the coexistence of denitrifying and anammox bacteria has been demonstrated and discussed. Anoxic conditions and influent C/N ratios lower than 2 are necessary conditions, among others, to stable achieve a denitrifying and anammox bacteria coexistence (Kumar and Lin, 2010).

The occurrence of nitritation, anammox and denitrification process in one single reactor has been named in literature as SNAD (Simultaneous partial Nitritation, Anammox, and Denitrification) process (Chen et al., 2009). It has been applied to treat both main and side-stream wastewaters (Daverey et al., 2012; Winkler et al., 2012). Most of the SNAD systems are operated in granular Sequencing Batch Reactors (SBRs), because both SBR technology and granulation allow the coexistence of several bacterial populations, facilitating a high biomass concentration, different environmental conditions (substrate stratifications) and the accumulation of slow growing organisms, such as nitrifying and anammox bacteria (Strous et al., 1998; De Kreuk and Van Loosdrecht, 2004; Li et al., 2011).

Moreover, both of these technologies improve the system resistance to shock and toxic loadings, preventing inhibition effects (Etterer and Wilderer, 2001; Mohan et al., 2005).

Furthermore, digester effluents could also contain substantial amounts of methane in addition to ammonium. In the last decade, other nitrogen converting bacteria have been discovered, such as the n-damo bacteria (Luesken et al., 2011b), capable of nitrite-dependent anaerobic methane oxidation. Coupled in a nitritation-anammox reactor, n-damo bacteria could be of interest for wastewater treatments as the overall system could be used to remove both methane and ammonium (Zhu et al., 2011).

A better understanding of the microbial community composition and concomitant knowledge of the physiology of the community members in a combined nitritation-anammox process treating digester effluents may lead to improved success for biological nitrogen removal.

Up to now, most studies have been focused on the diversity of AOB and anammox bacteria (Third et al., 2001; Vázquez-Padín et al., 2010; Wang et al., 2010). Limited works on the presence of denitrifiers in single nitritation and anammox reactors have been done, mainly looking at the influent and effluent COD and N levels and relate to their operation under aerobic/anoxic conditions. So far, there were only few studies on the microbial identification of the denitrifying bacteria in nitritation-anammox as well as in SNAD systems (Xiao et al., 2009; Keluskar et al., 2013). Moreover, the presence of n-damo bacteria have been shown in the activated sludge of WWTPs with relatively long sludge retention times (SRT) (Luesken et al., 2011b) and in a full scale anammox reactor (Zhu et al., 2011). Lacking more data, it remains unknown whether it will be the same or not in other bioreactors, performing the nitritation, and anammox process for treating digester effluents.

In this study, the biomass of a full scale application of a partial nitritation—anammox process in a SBR, treating digester supernatants, has been analyzed. The objectives of this study are (1) to evaluate the activity of AOB/anammox/denitrifing populations under different running conditions, by means of repeated batch-assays, in order to evaluate the optimal SBR set-up; (2) to investigate the diversity of AOB, anammox, as well as the denitrifiers community based on clone libraries of 16S rRNA and functional gene-based analyses; (3) to look toward the identification of other nitrogen converting bacteria in the nitritation-anammox biomass treating digester effluents.

## Materials and methods

### Reactor set-up

The nitritation-anammox sludge, investigated in this study, was collected from a running full scale SBR in Zürich (Switzerland), and previously described by Joss et al. (2009).

The SBR (1400 m^3^) treated the digester supernatant of the municipal WWTP, containing an average of 650 ± 50 mg NH4^+^-N/L and 300 ± 50 mg COD soluble/L. The SBR worked at high temperature (*T* = 30°C), under a nitrogen loading rate of 0.625 kg-N/m^3^/day, oxygen limiting conditions (DO< 0.8 mg/L). The pH varied during the SBR cycle between 7.0 and 7.5. In the running SBR, a complete nitrification- anammox process occurred (Joss et al., 2009), achieving high total ammonium (95%) and total nitrogen (94.6%) removal efficiencies. Further, a 37% organic matter removal efficiency has been reached, by means of carbon oxidation and denitrification processes.

The SBR cycle comprises a feeding phase, one or several aeration phases, one or several anoxic-mixing phases, a sedimentation phase, and a discharge phase; a pause of up to several days was intercalated between the discharge and the subsequent feeding phases to adapt to the incoming load. A complete cycle typically lasts between 6 and 24 h.

Under regular operations, the authors adopted several precautions to limit the growth of nitrite oxidizing bacteria (NOB): (i) maintain low substrate levels for nitrite oxidizers, keeping the oxygen concentration lower than 1 mg O_2_/L and allowing a maximum concentration of nitrite after the aeration step of 3–8 mg NO^−^_2_/L; (ii) work at high free ammonia (NH_3_-N) concentrations both at the beginning of the feeding step (reaching up to 200 mg NH^+^_4_-N/L in the reactor) and at the end of the SBR cycle (avoiding ammonia depletion completely, thus, keeping at least 10–40 mg NH^+^_4_-N/L); (iii) work at high temperature (*T* = 30°C). Further, the C/N ratio in the influent was around 0.5, preventing denitrifiers to outcompete anammox bacteria.

The sampled nitritation-anammox sludge contained granules surrounded by a matrix of brownish flocs. The granules had diameters between 0.1 and 2.0 mm.

### Activity analyses

Assays were performed to evaluate the AOB, anammox and denitrifying activity in the nitritation–anammox sludge.

Concentrated sludge was used for the batch assays. The protein concentration of the nitritation-anammox biomass was about 2.85 ± 0.42 mg protein/mL, while the total suspended solids content was 10 ± 0.5 gTSS/L, meaning that the protein concentration in the sludge was approximately a third of the measured dry weight (0.3 g protein/g biomass). A similar observation was previously reported for a CANON biomass (Third et al., 2001).

Sludge (10 mL) was washed 3–5 times with tap water in order to remove residual substrates. The last wash was performed using HEPES 20 mM as a buffer solution, at the desired pH. All incubations were performed at room temperature (*T* = 22 ± 1°C) and under continuous mixing (150 rpm). Activities were determined by measurement in batch tests of the rates of depletion of substrates. The values measured were then referred to the protein and biomass concentrations. To this end, measurements were performed as described below over a time span of 3 h with a sampling interval of 30 min.

#### AOB activity assays

The washed nitritation-anammox biomass (10 mL) was transferred to 30 mL conical glass flasks covered with a wad of cotton wool. To measure the aerobic ammonium oxidation activity, the flasks were incubated aerobically. Aerobic conditions were maintained under active mixing, ensuing a low and not limiting oxygen concentration (1.5 < DO < 2 mg/L). Substrate was added from a sterile 100 mM NH_4_Cl stock solution. First, batch tests were performed at the pH of the full-scale SBR at the beginning of the nitritation step (7.5), varying the initial concentrations of total ammonium nitrogen (TAN) (in the range of 1.5–13 mM), in order to investigate the limiting and inhibitory effects of TAN concentration on AOB. Then, in order to evaluate the overall effect of the reactor pH on the AOB activity, batch tests were performed using a constant initial TAN concentration of 9 mM and varying the pH (6.0, 6.5, 7.0, 7.5, 8.0, 8.5, 9.0, and 9.5).

#### Anammox activity assays

The washed SNAD biomass (10 mL) was transferred to 30 mL glass serum bottles. Bottles were sealed with butyl rubber stoppers and soluble substrates added from sterile 100 mM stock solutions (NH_4_Cl and NaNO_2_). Directly hereafter, the bottles were made anoxic by alternatingly applying under-pressure and flushing with N_2_ gas. An overpressure of 1 bar was maintained in the bottles. Batch tests were performed to investigate the short term effects of nitrite on the anammox activity: several initial TNN concentrations between 1.0 and 5 mM were tested at a constant pH of 7.5, working at not limiting TAN concentrations (4.0 mM), thus ensuring the theoretical anammox stoichiometry TNN/TAN ratio of 1.32. Then, in order to investigate the effect of the pH on the anammox activity, batch tests were performed using constant initial TAN (4.0 mM) and TNN (1.5 mM) concentrations and varying the pH (6.5–9).

#### Denitrifiers activity assays

The washed nitritation-anammox biomass (10 mL) was transferred to 30 mL glass serum bottles. Bottles were sealed with butyl rubber stoppers and soluble substrates added. The bottles were made anoxic by alternatingly applying under-pressure and flushing with N_2_ gas. An overpressure of 1 bar was maintained in the bottles. Batch tests were performed at pH 7.5. In order to evaluate denitrification via nitrate activity, into each bottle, nitrate from a sterile 10 mg NO^−^_3_-N/L stock solutions (KNO_3_), was added by means of needled syringes, achieving an initial 200 mg NO^−^_3_-N/L in 10 mL solution. Then, a flushed substrate solutions of CH_3_COONa (sterile 100 gCOD/L stock solutions) was supplemented. The initial amount of the carbon substrate corresponded to approximately 2000 mg COD/L. The same operation was repeated to evaluate denitrification via nitrite activity. Nitrite, from a sterile 10 mg TNN/L stock solutions (NaNO_2_), corresponding to an initial nitrite concentration of 200 mgTNN/L, was added followed by the addition of the carbon substrates as done in the tests with nitrate.

### Genomic DNA extraction and purification

To maximize DNA extraction efficiency a representative sample (0.3 mL) with granules and brownish flocs was taken and its granules and flocs were aseptically broken, by passing through a series of syringe needles (0.60–0.30 mm). Thereafter, DNA was extracted, using a CTAB DNA isolation method adapted from the protocol published by Zhou et al. (1996), and described previously (Yan et al., 2010).

### PCR amplification

PCR analyses were performed in a T gradient cycler (Whatman Biometra, Göttingen, Germany) using as buffer and enzyme formulation the GoTaq^®^ Green Master Mix (Promega Benelux BV, Leiden, the Netherlands), according to the manufacturer's protocol instructions. To analyze the microbial community composition in the nitritation—anammox process, independent PCR assays were conducted, targeting both the main (nitritation, anammox, and denitrification processes) and possible secondary biological processes (nitratation and n-damo processes). Amplifications, using primers targeting 16S rRNA genes (phylogenetic marker) and primers targeting genes coding for parts of physiologically relevant enzymes (functional marker), were performed for the relevant groups of microorganisms. The extracted genomic DNA was used as a template for PCR reactions targeting respectively: bacterial *amo*A genes which are diagnostic for AOB, *nxr*A genes diagnostic for *Nitrobacter* spp. NOB, *pmo*A genes diagnostic for methane-oxidizing bacteria and *nir*S genes diagnostic for denitrifying bacteria. In addition to functional gene targeting PCR reactions also 16S rRNA amplifications were performed using primers specific for *Nitrobacter* spp., *Nitrospira* spp., *Planctomycetes*, anammox and NC10 phylum bacteria (n-damo). Negative controls (no DNA added) were included in all sets of amplifications. Reaction mixtures (25 μL) used for PCR amplification contained 0.5 μL of each primer forward and 0.5 μL of each primer reverse, 12.5 μL of GoTaq^®^ Green Master Mix, including GoTaq^®^ DNA Polymerase, dNTPs, MgCl_2_ and reaction buffers, 1 μL of DNA diluted template corresponding to 25 ng of total DNA, and RNase-free water (DEPC) to complete the 25 μL volume. Specifications of all the primers used in this study are listed in the supplementary files (Table S1) including the annealing temperatures (*T*_*a*_). All PCR's were run with an initial denaturation of the template DNA at 96°C for 1 min, followed by 35 cycles of 1 min at 96°C, 1 min at a different annealing temperature (*T*_*a*_), and 1 min at 72°C. The reaction was completed after 10 min at 72°C. In all cases, PCRs were performed both using 1:100 and 1:10 dilutions of the original template DNA solution.

### Cloning and sequencing

Presence and size of amplicons were checked by agarose (1%) gel electrophoresis of 5 μL aliquots of the PCR products. PCR products were cloned directly using the pGEM-T Easy cloning vector kit, following the instructions of the manufacturer (Promega Benelux BV, Leiden, the Netherlands). Plasmid-DNA of clones was isolated from randomly selected clones per library using a GeneJET Plasmid Miniprep Kit (Fermentas GMBH, St. Leon-Rot, Germany). Clones were checked for inserts of the expected size by agarose (1%) gel electrophoresis after EcoRI digestion (5 U, Eco RI- buffer for 3 h at 37°C). Sequencing was performed at the DNA Diagnostics Center of Nijmegen University Medical Center, using the M13 forward primer.

### Nucleotide sequence accession numbers

The 16S rRNA and the functional gene sequences determined during this study were deposited in the GenBank database using the stand-alone software tool Sequin developed by the NCBI (http://www.ncbi.nlm.nih.gov/Sequin/). The partial *amo*A gene sequences are available under the accession numbers KC569470–KC569477 (*Nitrosomonas* spp.), the partial *nir*S gene sequences are found under KC569496–KC569503 (denitrifiers), while the partial 16S rRNA gene sequences are available under the accession number KC569478–KC569483 (anammox species) and KC569484–KC569495 (*Nitrobacter* spp.). Due to the simultaneous occurrence of nitritation, anammox and denitrification (SNAD) process, in this study the clone sequences found in the SBR were named by using the suffix SNAD.

### Phylogenetic analysis

Clone gene sequences were analyzed by BLASTN searches (http://blast.ncbi.nlm.nih.gov/Blast.cgi). The clone sequences were aligned using the MEGA5 software (Tamura et al., 2011). Sequences of related species and a selection of relevant clone sequences from different environments taken from the Genbank database were also included in the alignments. Phylogenetic trees were constructed based on nucleic acid (16S rRNA, *amo*A, *nir*S) and amino acid (AmoA, NirS) sequence alignments using the same software, applying the neighbor joining statistical method (Saitou and Nei, 1987) with the pairwise deletion option for gaps. The tree topology was based on bootstrap analysis. Evolutionary distances were computed using the Jukes-Cantor substitution model (Jukes and Cantor, 1969) for nucleic acid analysis. Poisson correction model (Zuckerkandl and Pauling, 1965) and the Dayhoff matrix based method (Schwarz and Dayhoff, 1979) were used for amino acid analysis.

### Analytical methods

Total suspended solids (TSS) were quantified weighing samples after drying at 105°C for 24 h and subsequent cooling in a desiccator. The Biuret method was used to determine the protein concentration (Layne, 1957). Samples of the activity assays (1 mL) were centrifuged (5 min, 10,000 × g) and the resulting supernatants analyzed for ammonium, nitrite and nitrate concentrations. Ammonium, as total ammonium nitrogen (TAN), and nitrite, as total nitrite nitrogen (TNN), were measured colorimetrically at 420 nm (adapted from Taylor et al., 1974) and 540 nm (adapted from Griess-Romijn van Eck, 1996), respectively. Nitrate as nitrogen (NO^−^_3_-N) and chemical oxygen demand (COD filtrate), were measured according to the Standard Methods (APHA-AWWA-WPCF 2005).

Concentrations of free ammonia (FA or NH_3_-N) were calculated using the equation Eqs. 1, derived from the acid—base equilibrium, as a function of pH, temperature and the sum of unionized and ionized forms (Anthonisen et al., 1976)

(1) $$FA[mgNL^{-1}]=\frac{TAN}{1+(10^{-pH}/e^{-6344/(273+T)})}$$

## Results

### Activity assays

#### Aerobic ammonia oxidizing bacteria

The AOB batch tests showed the limiting effect of TAN concentration on the AOB activity at TAN of 4 mM (56 mg TAN/L), corresponding to 0.8 mg NH_3_– N at pH 7.5. The maximum AOB activity was measured at 9–13 mM TAN (126–182 mg TAN/L), that is the maximum concentration of TAN in the reactor, at the beginning of the SBR cycle. At TAN concentrations of 9 and 13 mM and pH of 7.5, the AOB activity reached about 1.6 nmol N/mg protein/min, corresponding to about 3.8 mg TAN/gTSS/h. The equivalent free ammonia concentrations were 1.9–2.8 mg NH_3_– N/L, respectively. In the AOB activity tests, as total ammonium was consumed nitrite was produced and accumulated in the tests, resulting in a molar ratio of TNN_produced_/TAN_removed_ of 1, in accordance to the nitritation stoichiometry performed by AOB.

**Figure 1:**
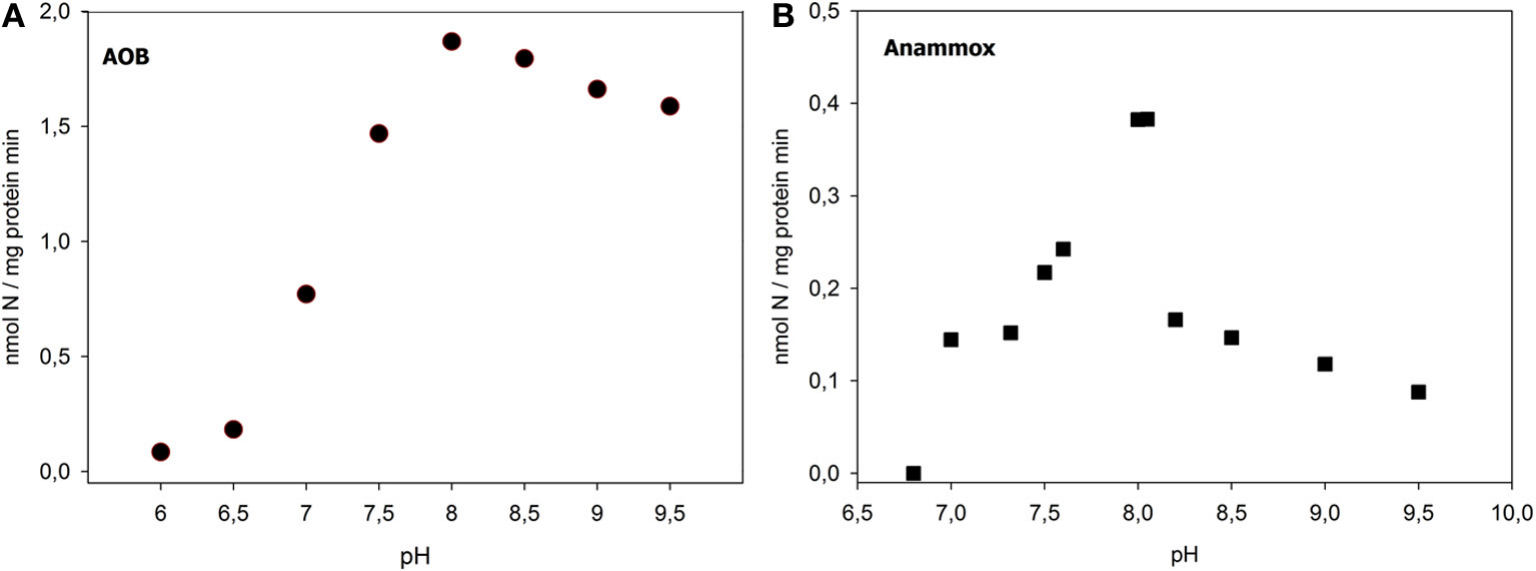
**pH effect on AOB (A) and anammox (B) activity in the nitritation-anammox sludge**.

When activity assays were performed at different pH, we found the optimal pH of 8 for AOB activity (1.86 nmol N/mg protein/min), as also reported by previous studies (Alleman, 1984) (Figure 1A).

#### Anaerobic ammonia oxidizing bacteria

The anammox activity at pH of 7.5 reached the maximum value at a TNN concentration of 3 mM, corresponding to 42 mg TNN /L (0.67 nmol N/mg protein/min), while started to decrease for higher nitrite concentrations.

Concerning the pH effects, according to Strous et al. (1997), we found an optimal pH of 7.8–8.0 for anammox activity (Figure 1B), for which a maximum potential activity of 0.38 nmol N/mg protein min, corresponding to 0.82 mg TAN/gTSS h, was measured. In the batch assays at pH of 7.8 and 8.0, the calculated molar ratios of TNN_removed_/TAN_consumed_ of anaerobic ammonia oxidation were higher than the theoretical anammox stoichiometry (1.32), equal to 1.4 and 1.6, respectively.

#### Denitrifying bacteria

Both nitrate and nitrite heterotrophic denitrification routes were measured in the activity assays, reaching a maximum activity of 6.25 mgNO^−^_3_-N_removed_/gTSS/h and 3.47 kgTNN_removed_/kgTSS/h, respectively. We also observed that in the activity assays specific for denitrification via nitrate route, nitrite accumulation occurred for the 1st 5 h. After 24 h nitrite were completely consumed.

### Phylogenetic analyses and microbial community of the nitritation - anammox sludge

#### Aerobic ammonia oxidizing bacteria

**Figure 2:**
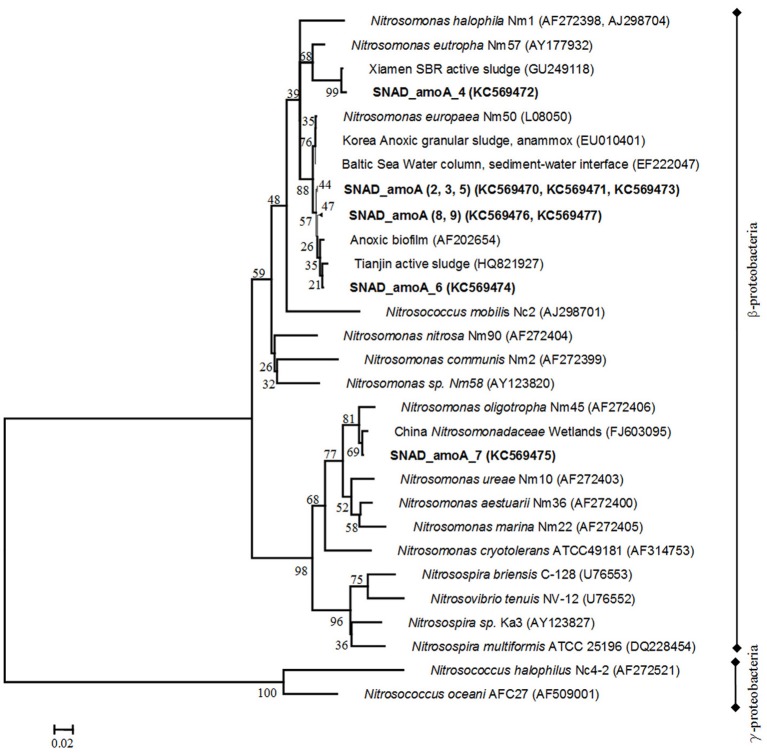
**Phylogenetic tree, reflecting *amo*A gene diversity.** Names of AOB are printed in italics and names of clone sequences are printed in bold. The phylogenetic tree was constructed using the Neighbour—Joining method with the Poisson correction model. Distances are in the units of the number of amino acid substitutions per site. The analysis involved 32 amino acid sequences. Evolutionary analyses were conducted in MEGA.5. The numbers at the nodes indicate the levels of bootstrap support (based on data for 500 replicates) for groups to the right of the nodes. The scale bar represents 2% estimated sequence divergence.

Specific primers (AmoA-1F/AmoA-2R) for the subunit A of the ammonium monooxygenase (*amo*A) enzyme, key enzyme in aerobic ammonium oxidation, were used to detect AOB. Correct-sized products of 453 nt were obtained. After transformation and cloning, eight clones were randomly selected for plasmid DNA isolation and sequencing. Phylogenetic analysis of amino acid sequences, deduced from the DNA sequences, showed that the AOBs in the samples were affiliated to environmental clone sequences from wastewaters and other engineered nitrogen treatment systems. Clone sequences, closely related to different members of genus *Nitrosomonas* (*N. europaea, N. eutropha*, and *N. oligotroph*a), were detected, suggesting a high AOB species diversity in the nitritation-anammox sludge (Shannon diversity index: *H* = 0.735, *E*_*H*_ = 3538) (Figure 2). As expected on the basis of the high total ammonium load (0.625 kgTAN/m^3^ d) of the full scale SBR (Joss et al., 2009), six clones were closely related to *Nitrosomonas europaea* (L08050, 97–98% similarity). The clone sequences were highly similar to environmental sequences previously recovered in an anoxic granular sludge (EU010401), an anoxic biofilm (AF202654) and an activated sludge, treating ammonium rich wastewaters (HQ821927). Similar results were observed by Pynaert et al. (2003) in a highly loaded Rotating Biological Contactor (RBC). We also observed one clone (SNAD_amoA_4) belonged to the *Nitrosomonas-eutropha*-like cluster (AY177932, 100% identity), usually detected and demonstrated to have activity in oxygen-limited environments (Li et al., 2009). Both *Nitrosomonas europea* and *Nitrosomonas eutropha* are capable of tolerating high-ammonia concentration (Holmes et al., 1995; Szatkowska et al., 2007). Finally, the sequence SNAD_AmoA_7 was distantly related to all of the previously recognized sequences and closely linked to the *Nitrosomonas-oligotropha*-like cluster (AF272406, 92% identity), which have been found usually in pools with low ammonia/ammonium concentration (Park et al., 2002). The clone sequence was extremely similar to environmental sequences previously recovered in a constructed wetland for wastewaters treatment (FJ603095).

#### Anaerobic ammonia oxidizing bacteria

**Figure 3:**
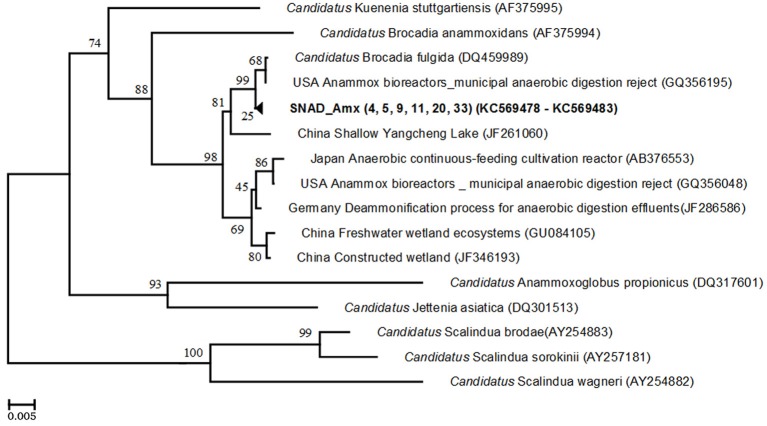
**Phylogenetic tree, based on 16S rRNA gene sequences using anammox-specific primers.** The tree shows the phylogenetic position of the clone sequences within the anammox bacterial genera. The clone sequences are in bold. Names of anammox species are printed in italics. The phylogenetic tree was constructed using the Neighbour—Joining method with the Jukes-Cantor model. The analysis involved 21 nucleotide sequences. Evolutionary analyses were conducted in MEGA.5. The numbers at the nodes indicate the levels of bootstrap support (based on data for 500 replicates) for groups to the right of the nodes. The scale bar represents 0.5% estimated sequence divergence.

To determine the diversity of anammox bacteria, two different primer combinations were used. The *Planctomycete* 16S rRNA was amplified using *Pla46F* and *630R* primers and correct-sized (1483 nt) products cloned. Nine clones were randomly selected for sequencing and almost full-length 16S rRNA sequences (average length 1490 bases) were obtained. Subsequent phylogenetic analysis showed that the clones were associated with the phylum Planctomycetes but none of the sequences were similar to the known anammox bacteria. When the anammox specific primer combination (368F—820R) was applied, a clear PCR product of 452 bp was obtained. Six clones were randomly selected and sequenced. Phylogenetic analysis of the nucleotide sequences revealed that the clone sequences were highly similar to each other (more than 99% sequence similarity), forming the cluster SNAD_Amx (4, 5, 9, 11, 20, and 33). Bootstrap analysis confirmed that this SNAD-Amx cluster was closely related (99%) to the known anammox bacteria “*Candidatus* Brocadia fulgida” (99% similarity) (Figure 3). The cluster was most closely related to clones retrieved from a USA anammox reactor, treating anaerobic digestion effluents (GQ356195).

#### Denitrifying bacteria

**Figure 4:**
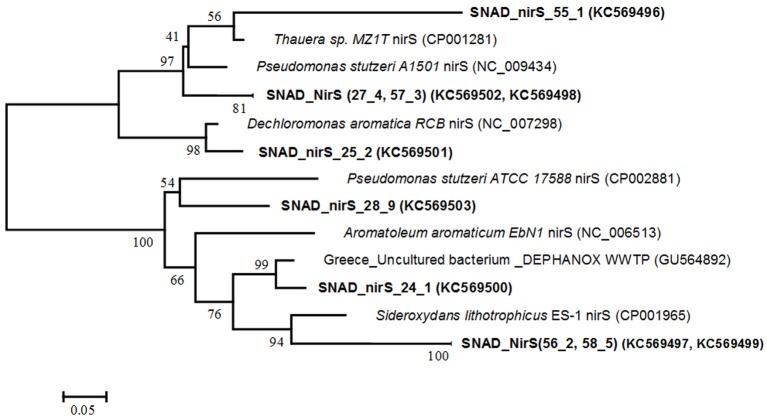
**Phylogenetic tree, based on nitrite reductase (*nir*S) functional gene sequences obtained with the nirS1F/6R primers.** Names of denitrifiers are printed in italics, while the *Nir*S clones are printed in bold. The phylogenetic tree was constructed using the Neighbour—Joining method with the Dayhoff matrix based method. The analysis involved 14 amino acid sequences. Evolutionary analyses were conducted in MEGA.5. The numbers at the nodes indicate the levels of bootstrap support (based on data for 500 replicates) for groups to the right of the nodes. The scale bar represents 5% estimated sequence divergence.

The diversity of denitrifiers was assessed by using the dissimilatory nitrite reductase (*nir*S) gene as a proxy and the NirS1-NirS 6 primers combination. The correct-sized (875 nt) amplificated were cloned and eight clones were randomly selected and further analyzed by comparative sequencing. The phylogenetic tree of nitrite reductase genes (*nir*S) is presented in Figure 4. Phylogenetic analysis revealed the clone sequences were related to the *nir*S sequences from different anaerobic denitrifying bacteria, such as *Thauera* spp., *Pseudomonas stutzeri*, *Dechloromonas aromatica* and *Aromatoleum aromaticum*, which have been shown to degrade, among the others, several aromatic compounds and to be involved in the granular/biofilm formation process. Further, the sequence SNAD_nirS 24 was closely related to clones from a biological anoxic phosphorus removal reactor (DEPHANOX-type - GU564892) where denitrifying polyphosphate accumulating organisms (DPAOs) have been observed. Finally, the cluster SNAD_NirS (56.2, 58.5) was 88% similar to the lithoautotrophic microaerophilic Fe(II)-oxidizing betaproteobacterium *Sideroxydans lithotrophicus* ES-1 (CP001965), which may play a role for Fe(II) oxidation in wastewaters containing Fe(II).

#### Possible secondary biological processes

**Figure 5:**
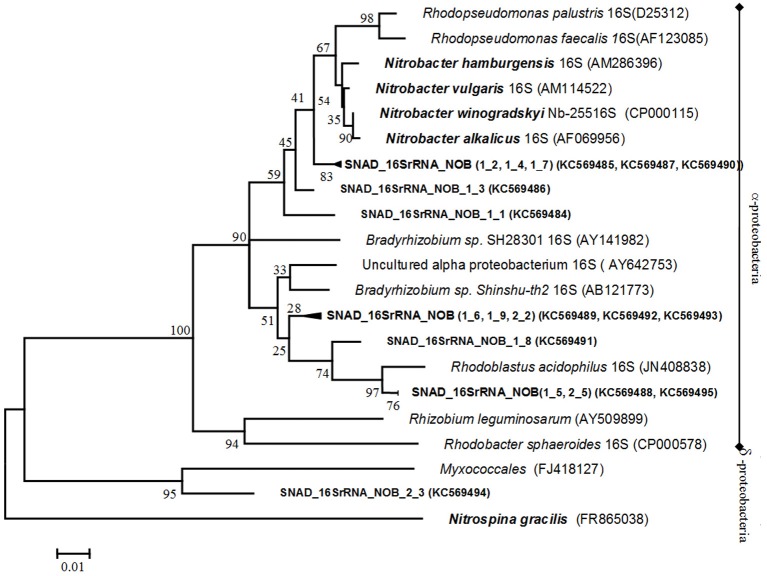
**Phylogenetic tree, based on 16S rRNA gene sequences of NOB.** The tree shows the relationship of the clone sequences within the α- and δ- protobacteria, including both nitrite-oxidizing and non-nitrifying bacteria. Names of nitrite oxidizers are printed in bold and italics while clone sequences are in bold. The phylogenetic tree was constructed using the Neighbour—Joining method while the distances were computed using the Jukes-Cantor method. The analysis involved 26 nucleotide sequences. Evolutionary analyses were conducted in MEGA.5. The numbers at the nodes indicate the levels of bootstrap support (based on data for 500 replicates) for groups to the right of the nodes. The scale bar represents 1% estimated sequence divergence.

Bacteria involved in the possible secondary biological processes such as NOB and n-damo bacteria were investigated. First, the NOB diversity was analyzed by using multiple primer sets. The primer combination Nitro1198F/Nitro1423R, targeting the 16S rRNA genes of *Nitrobacter*-like bacteria (Graham et al., 2007), yielded products of the correct size (225 nt), while no PCR products were obtained using primers targeting the *nxr*A gene of *Nitrobacter* spp., nor the 16S rRNA gene of *Nitrospira* spp. The PCR, targeting the 16S rRNA gene of *Nitrospira* spp., did not yield any specific products, which may indicate either that the *Nitrospira* spp. cell numbers are too low for PCR detection or that a *Nitrospira* species not targeted by the primer set are present. A 16S rRNA gene clone library was constructed from the aggregate samples to identify the nitrite oxidizers in the SNAD sludge. Twelve clones were selected at random and used for comparative sequence analysis. The NOB phylogenetic analysis is presented in Figure 5. The 16S rRNA sequences of two of the twelve clones (SNAD_16SrRNA_NOB_1_1), (SNAD_16SrRNA_NOB_1_3) and the cluster SNAD_16SrRNA_NOB(1_2, 1_4,1_7) were related to the members of nitrite oxidizers of the *Nitrobacter hamburgensis* (98% identity) and *Rhodopseudomonas* (99% identity). Recent research reported the ability of non-nitrifying bacteria related to the *Rhodopseudomonas* spp. (purple non-sulfur bacteria -strain LQ17) to use nitrite phototrophically as an electron donor as well (Schott et al., 2010). In addition, in our study, five of the remaining clone sequences (SNAD_16SrRNA_NOB(1_5, 2_5), SNAD_16SrRNA_NOB(1_6, 1_9, 2_2) and SNAD_16SrRNA_NOB_1_8) were closely related to *Nitrobacter* spp. closest non-nitrifying relatives, *Bradyrhizobium* sp. *Shinshu-th2* and *Rhodoblastus acidophilus*. The detection of non-nitrifying relatives may be explained considering both the low phylogenetic diversity of *Nitrobacter* spp. (Daims et al., 2011) and their high 16S rRNA similarities to their closest non-nitrifying relatives. Some studies reported that the 16S rRNA method seems to be too conserved to unequivocally determine evolutionary lineage within the genus *Nitrobacter* (Daims et al., 2011). The remaining partial sequence (SNAD_16SrRNA_2_3) was related to the 16S rRNA gene of a non–nitrifying bacterium (*Myxococcales*) of the deltasubclass of proteobacteria.

Finally, as dissolved methane from anaerobic digester reactor can be carried over to the nitritation-anammox reactor treating anaerobic digester supernatants, the n-damo bacteria (Ettwig et al., 2009) were also screened in our study, using both the 16S rRNA and the functional level approach (Luesken et al., 2011b). In order to detect “*Ca*. Methylomirabilis oxyfera,” first a direct PCR with *pmo*A primers was applied. PCR products were not of the expected size and therefore not further analyzed for sequencing. The results indicate that either the amount of n-damo bacteria in the nitritation-anammox sludge were too low for PCR detection or that the primer combination used for the direct PCR was not targeting the n-damo bacteria present in the sludge (Luesken et al., 2011a). The nested PCR approach and the sequences retrieved with this did not yield any n-damo bacterial sequences either. The clones clustered with an uncultured alpha proteobacterium from environmental DNA sampled from oligotrophic environments (obtained from 15 m depth in the Northern Atlantic Gyre) (AM706683—100% identity).

## Discussion

In this study, the activities of AOB, anammox and denitrifying bacteria in a nitritation—anammox biomass were studied in order to evaluate the operative conditions which allow an overall stable nitritation—anammox process for treating anaerobic digester effluents. Furthermore, the microbial community composition of the sludge was analyzed, looking at the main as well as the possible secondary biological processes.

First, the AOB activity and its equilibrium with NOB activity are discussed. Previous studies in literature clearly show AOB activity is influenced by the TAN concentrations, and, better, by the corresponding free ammonia (NH_3_) concentration, because NH_3_, rather than NH^+^_4_, acts both as substrate and inhibitors for AOB (Suzuki et al., 1974). Further, NH_3_ acts as inhibitor for NOBs (Anthonisen et al., 1976). In particular, AOB are inhibited at NH_3_– N concentrations between 10–150 mg/L, while inhibition of NOB is observed at lower NH_3_– N concentrations, in the range of 0.08–0.82 mg/L (Anthonisen et al., 1976). In this study, we showed that, operating at pH of 7.5 and using the minimum (4 mM TAN) and the maximum (13 mM TAN) ammonium working conditions of the full-scale SBR, the free ammonia level in the reactor was always not inhibiting for AOB (lower than 10 mg NH_3_-N/L). On the contrary, the free ammonia level was inhibiting for NOB (higher than 0.08–0.8 mg NH_3_-N/L). Thus, the nitritation-anammox SBR, working at pH of 7.5 at the beginning of the nitritation step, was able to reduce the NOB activity by ensuring high TAN value (9–13 mM TAN) at the beginning of the SBR and further by avoiding the complete TAN depletion at the end of the SBR cycle. This observation was further demonstrated looking at the TNN_produced_/TAN_removed_ ratio in the AOB assays, which has been always about 1, demonstrating the low activity of NOB in the nitritation-anammox sludge sampled and confirming that the operating conditions applied to the SBR were able to reduce the NOB activity in the nitritation –anammox process.

Furthermore, in this study, we also evaluate the optimal working pH for AOB activity. As far as we know, the impacts of the pH on AOB activity are both direct (linked to the enzymatic activity) and indirect (associated with nutritional effects), and it is difficult to distinguish them. In this study the overall pH effect is considered. Working with an initial TAN concentration of 9 mM in the batch assays, an optimal pH of 8.0 has been found. The corresponding free ammonia level was 5.9 mg NH_3_-N/L. This value is a non limiting and non-inhibiting substrate concentration for AOB, while is inhibiting for NOB. Thus, in order to increase the AOB activity in the SBR, a possibility would be to operate the reactor at pH values of 8.0, higher than the value reported by Joss et al. (2009).

According to the phylogenetic analysis, our results supported the hypothesis that in the nitritation-anammox process, carried out in an SBR, different macro- and micro-environments strongly affected the AOB population structure. The different oxic and anoxic phases and the presence of dense biomass-flocs and granules can create physical and chemical gradients of substrates. The high ammonia concentration in the bulk liquid at the beginning of the SBR cycle is a critical factor for the involvement of *Nitrosomonas europea* and *Nitrosomonas eutropha* relatives in the flocs and in the outer part of granules, while the low ammonia concentrations in the inner part of granules and in the bulk liquid at the end of the SBR cycles allow the development of *Nitrosomonas-oligotropha*-spp. The high diversity of AOB in the nitritation-anammox-denitrification process found in this study is completely in congruence with earlier reports, where nitrifiers coexist together with anammox and denitrifying bacteria in granular or biofilm systems (Xiao et al., 2009; Keluskar et al., 2013). The high AOB species diversity may be beneficial to the stability of wastewater treatments (Daims et al., 2001) and in particular for the nitritation-anammox and SNAD process.

Concerning the anammox bacteria, we found that short term exposure of the nitritation-anammox biomass to nitrite levels up to 3 mM TNN (42 mg/L) did not result in inhibition of the anammox bacteria. The inhibiting nitrite concentration measured in this study is much higher than the maximum nitrite concentration reached in the reactor at the beginning of the anoxic phase of the SBR cycle, when anammox process start. This indicated that 3–8 mg TNN/L, reached at the end of nitritation process in the full scale SBR, is a safe level of nitrite concentration, avoiding anammox inhibition and furthermore NOB proliferation, due to substrate limitation for NOB. Furthermore, in order to increase the anammox activity in the reactor, we also found that could be possible to work in the SBR at a pH of 7.8–8.0, higher values than the value used by Joss et al. (2009).

The TNN_consumed_/TAN_consumed_ ratios measured in this study were higher than 1.32, indicating that other pathways, that involve nitrogen removal, occurred in the nitritation-anammox process. A part of the nitrite may be converted by heterotrophic denitrifiers (Kumar and Lin, 2010) or “disguised” anammox bacteria (Kartal et al., 2007; Kartal, 2008), which may use organic carbon substrates present in the sludge, mainly cell lysis products. Indeed, heterotrophic denitrifiers could use organic carbon substrates present in the sludge to reduce nitrite (Kumar and Lin, 2010), leading to a higher nitrite consumption and an overestimation of the TNN/TAN ratio. Further, an alternative anammox pathway may occur. Anammox bacteria are able to use a range of organic electron donors and inorganic electron acceptors. If nitrate or nitrite are present, short chain fatty acids, (i.e., acetate, formate, propionate) can be metabolized by anammox bacteria, capable of dissimilatory nitrate reduction to ammonium (DNRA), reducing NO^−^_3_ or NO^−^_2_ to NH^+^_4_, that could be used consequently for the traditional anammox pathway producing N_2_ gas (Kartal et al., 2007; Kartal, 2008). This alternative anammox pathway would lead to an ammonium production, thus to an underestimation of the ammonia consumed in the anammox process and, again, an overestimation of the TNN/TAN ratio. In the anammox activity assay at pH 7.8 and 8, we evaluate the classical anammox process (anaerobic ammonium oxidation) contributed up to 95% and 80% respectively of total nitrite consumption while the other processes, denitrification and/or alternative anammox pathway, overall account for 5% and 20%.

The phylogenetic analysis showed that “*Ca*. Brocadia fulgida” was the dominant anammox bacterium. Our results were in line with previous studies that found “*Ca*. Brocadia” as the abundant anammox species in WWTPs, where organic compounds, together with ammonium, nitrite, and nitrate, are present (Hu et al., 2010), out-competing other anammox bacteria (Kartal et al., 2008). Treating landifil leachate by a a sequencing batch biofilm reactor (SBBR) at pH of 8.1–8.5 and oxygen limiting conditions, Xiao et al., 2009 found “*Ca*. Brocadia anammoxidans” in their reactor. The different anammox bacteria could be due to the different influent wastewater and the different operative conditions. However, the identification of the anammox bacterial genera is a useful information to operate the SBR reactor, i.e., avoiding nitrite inhibiting concentration. *Ca*. Brocadia are able to tolerate up to 42 mgTNN/L (Kartal et al., 2008), confirming, thus, the right operative conditions of the full-scale SBR, which worked with a maximum nitrite concentration of 3–8 mgTNN/L at the beginning of the anoxic phase of the SBR.

Further, in the full scale nitritation-anammox reactor, treating digester supernatant with a C/N ratio of 0.5, we measured the denitrification activity. Both reduction of the nitrate and nitrite occurred, using acetate as substrate in the activity assays. The results suggested denitrifying bacteria in nitritation-anammox process can compete with anammox for nitrite consumption during the anoxic phase and furthermore can reduce nitrate produced by anammox process, improving the total nitrogen removal process. Nitrate and nitrite reduction was also observed in a full scale partial nitritation and anammox reactor (Desloover et al., 2011).

In this study, phylogenetic analysis of the deduced amino acid sequences revealed that the nitritation-anammox biomass was characterized by a high diversity of denitrifiers of the betasubdivision of the proteobacteria, showing the importance of denitrification process in the nitritation-anammox systems, treating ammonium-rich wastewaters which contain organic substrate. The importance of denitrifying bacteria in a nitritation-anammox process was related to the overall nitrogen removal efficiencies, contributing to remove nitrate produced by the anammox process (Desloover et al., 2011), but also to other aspects, such as aggregate formation. Most of the denitrifiers detected in the present study are reported to facilitate the formation of aggregates thereby preventing the biomass from getting washed out from the reactor. *Thauera* MZ1T is unique among the *Thauera* spp. in its production of abundant extracellular polymeric substances (EPS), which may contributes to the granular process (Heylen et al., 2006), justifying their presence in the suspended-growth/granular biomass of the full scale SBR. *Dechloromonas aromatica* strain RCB appears to support a highly complex lifestyle which might involve biofilm/granules formation and interaction with an eukaryotic host (Salinero et al., 2009). Similar conclusion was presented by Keluskar et al. (2013) in a granular SNAD process. They detected, using *nir*S and *nos*Z genes, denitrifiers belonged to alpha (*Rhodopseudomonas* sp.), beta (*Thauera* sp., *Pusillimonas* sp., *Acidovorax* sp., *Comammonas* sp.) and gamma (*Thermomonas fusca*, *Xanthomonas* sp.) proteobacteria. Most of them are able to carry out simultaneous heterotrophic nitrification–denitrification process and to assist in forming microbial aggregates. In this study, other denitrifiers, with specific functions, were identified, such as the *Pseudomonas stutzeri* and *Dechloromonas aromatic*, which both encode proteins suggestive of the ability to fix nitrogen and CO_2_ (Yan et al., 2005; Salinero et al., 2009). Further, the presence of denitrifying polyphosphate accumulating organisms (DPAOs) is probably mirrored by the fact that in the SBR anaerobic/anoxic and aerobic phases alternate, favoring phosphorus release/uptake. This indicates that also phosphorus removal process could play a role and should be investigated in nitritation/anammox-type reactors. Xiao et al. (2009) also showed a great denitrifiers diversity in their SBBR performing the SNAD process. They identified three different anaerobic denitrifying bacteria, *A. delafieldii* of band P, B. *clausii* of band Y and *C. variabile* of band b, and an aerobic denitrifying bacterium, *Hyphomicrobium* sp. of band D which transformed nitrate to nitrite.

Finally, concerning the possible secondary biological processes involved in the nitritation-anammox process, phylogenetic analysis showed the presence of NOB and the absence of n-damo bacteria. Although recent metagenomic-based studies (Daims et al., 2011) indicated that *Nitrospira*-like bacteria are more abundant NOB in sewage treatment systems, our PCR analysis showed that *Nitrobacter*-like bacteria, rather than *Nitrospira* spp., were the dominant NOB in the nitritation-anammox biomass. Nevertheless, the detection, in the nitritation-anammox sludge, of only *Nitrobacter* spp. is in agreement with results of previous studies, where significant amounts of *Nitrobacter* spp. have been detected by FISH in high-loaded WWTPs (Mobarry et al., 1996; Gieseke et al., 2003). Compared to *Nitrospira* spp., *Nitrobacter* spp. are inhibited at higher free ammonia concentrations (Kim et al., 2008). Further, *Nitrobacter* spp. have been reported to be less sensitive to free nitrous acid than *Nitrospira* spp. (Blackburne et al., 2007).

In their SBBR performing the SNAD process, Xiao et al., 2009 detected NOB of the genus *Nitrobacter* spp., *Nitrospina* spp. and *Nitrospira* sp. The authors explained that the abundant species diversity of NOB could be linked to the fact that the AOB offered most of the nitrite needed in the reactor. However, in the full scale nitritation—anammox SBR, the presence of NOB in the reactor does not seem to compromise the nitrogen removal efficiency, probably due to low NOB activity caused by the selective pressures in the Zürich SBR management (low dissolved oxygen, high free ammonia concentration, high temperature). The low NOB activity in the sludge has also been confirmed by the AOB activity assays, where no nitrite was further oxidized to nitrate. Nevertheless, under inappropriate reactors operative conditions, NOB may be the cause of instability of the nitritation-anammox process. In this case, both the identification of NOB and their quantification, through qPCR analysis, may be useful tools in setting-up the reactor operative parameters (pH, temperature, free ammonia and free nitrous acid concentration levels) and in preventing instability, due to the NOB proliferation, in nitritation-anammox working reactors.

In contrast, no n-damo bacteria were detected in the nitritation-anammox sludge. The results may indicate that they were not sufficiently abundant to be detected (Zhu et al., 2011), and further analysis would be required to definitively prove the absence of these microorganisms in the sludge.

In this study we have got some interesting findings that a high species diversity in the microbial community of the nitritation-anammox reactor contributed to the overall nitrogen removal process. Further, the identification of specific groups of bacteria, and the study of their activity may contribute to a better definition of design criteria, and enable stable operations of complicated but challenging biological processes.

Nevertheless, this study is a step forward in understanding of the overall microbial community composition of the community members in a combined nitritation-anammox process treating digester effluents. The diversity of heterotrophic denitrifiers may be evaluate by using different functional genes or enzymes, involved in denitrification as molecular markers, looking at both the nitrite and nitrate reduction routes. Further research, e.g., by designing and using less specific primers, is needed to conclusively validate the absence of *Nitrospira* spp and n-damo bacteria bacteria.

### Conflict of interest statement

The authors declare that the research was conducted in the absence of any commercial or financial relationships that could be construed as a potential conflict of interest.

## Supplementary materials

Table S1 **Primers used for PCR amplifications and sequencing**.
