# Supplementary material for: Coexistence of nitrifying, anammox and denitrifying bacteria in a sequencing batch reactor
Source: Front Microbiol. 2014 Feb 4;5:28. doi: 10.3389/fmicb.2014.00028 (PMC3912432; doi:10.3389/fmicb.2014.00028)
Supplement: Table S1 — Primers used for PCR amplifications and sequencing. [file Data_Sheet_1.pdf]

Coexistence of nitrifying, anammox and denitrifying bacteria in a sequencing batch reactor

Michela Langone[1], Jia Yan[2,3], Suzanne C.M. Haaijer[2], Huub J. M. Op den Camp[2], Mike S. M. Jetten[2], Gianni Andreottola[1]

[1] Department of Civil, Environmental and Mechanical Engineering, University of Trento, Trento, Italy
[2] Department of Microbiology, Institute for Water and Wetland Research, Radboud University Nijmegen, Nijmegen, Netherlands
[3] Department of Environmental Engineering, College of Environment and Energy, South China University of Technology, China

Corresponding author:
Michela Langone,
Department of Civil, Environmental and Mechanical Engineering,
University of Trento,
Via Mesiano 77,
Trento, 38123,
Italy.
e-mail: michela.langone@ing.unitn.it, langonemichela@gmail.com

List of Supplementary Files

Table S1. Primers used for PCR amplifications and sequencing

Table S1. Primers used for PCR amplifications and sequencing

Name primer	target organism(s)	target gene	sequence (5'-3') of primer	Position	T_a (°C)	Reference
**AmoA-1F**	*Nitrosomonas* spp.	*amoA*	GGGGTTTCTACTGGTGGT	332-349 [a]	52	(Rotthauwe et al., 1997)
**AmoA-2R**			CCCCTCKGSAAAGCCTTCTTC	802-822 [a]		(McTavish et al., 1993)
**Pla46-F**	*Planctomycetes*	16S	GGATTAGGCATGCAAGTC	46-63 [a]	56	(Neef et al., 1998)
**630 R**	Most Bacteria		CAKAAAGGAGGTGATCC	1529-1545 [a]		(Schmid et al., 2003; Juretschko et al., 1998)
**Amx 368F**	*Anammox bacteria*	16S	CCTTTCGGGCATTGCGAA	368-385	56	(Schmid et al., 2003)
**Amx 820R**	*Kuenenia/ Brocadia*		AAAACCCCTCTACTTAGTGCCC	820-841 [b]		(Schmid et al., 2000, 2003)
**NirS1F**	*Denitrifiers*	*nirS*	CCTA(C/T)TGGCCGCC(A/G)CA(A/G)T	763-780 [b]	57	(Braker et al., 1998; Throbäck et al., 2004)
**NirS6R**			CGTTGAACTT(A/G)CCGGT	1638-1653 [b]		(Braker et al., 1998; Throbäck et al., 2004)
**Nitro-1198F**	*Nitrobacter* spp.	16S	ACCCCTAGCAAATCTCAAAAAACCG	1198[a]	58	(Graham et al., 2007)
**Nitro1423R**			CTTCACCCCAGTCGCTGACC	1423[a]		(Graham et al., 2007)
**NxrA F1370 F**	*Nitrobacter* spp.	*nxrA*	CAGACCGACGTGTGCGAAAG	1370	55	(Wertz et al., 2008)
**F2843 R**			TCCACAAGGAACGGAAGGTC	2843		(Wertz et al., 2008)
**Nspra-675F**	*Nitrospira* spp. (total)	16S	GCGGTGAAATGCGTAGAKATCG	675–696 [a]	58	(Graham et al., 2007)
**Nspra-746 R**			TCAGCGTCAGRWAYGTTCCAGAG	746–768 [a]		(Graham et al., 2007)
**Ntspa 616F-**	*Domain Bacteria*	16S	AGAGTTTGATYMTGGCTC	8-25 [a]	56	(Juretschko et al., 1998)
**Ntspa 1158R**	*Nitrospira* spp.		CCCGTTMTCCTGGGCAGT	1158–1176[a]		(Maixner et al., 2006)
**PmoA-A47F**	*n-damo* bacteria	*pmoA*	TCACGTTGACGC CGA TCC	47	60	(Luesken et al., 2011)
**PmoA-A682R**			GAAASGCNGAGAAGAASGC	682		(Holmes et al., 1995)
**202F**	*'N10' Phylum bact.*	16S	GACCAAAGG GGGCGAGCG	193 [c]	57	(Ettwig et al., 2009)
**1543R**	Most Bacteria		TCTCCACGCTCCCTTGCG	1027 [c]		(Juretschko et al., 1998)
**qP1F**	*M. oxyfera*		GGGCTTGACATCCCACGAACCTG	1016-	65	(Ettwig et al., 2009)
**qP2R**	Most Bacteria.		CTCAGCGACTTCGAGTACAG	1481		(Ettwig et al., 2009)
**M13 Forward**	Sequencing	pGEM T easy vector	GTAAAACGACGGCCAG	Region flanking cloning site	-	-

[a] *Escherichia coli numbering* (Brosius et al., 1981).
[b] Positions in the *nirS* gene of *Pseudomonas stutzeri* ZoBell EMBL (X56813).
[c] Positions based on the gap-free sequence of the N10 bacterial clone D-BACT (DQ369742), starting at *Escherichia coli* position 8.

Reference

Braker, G., Fesefeldt, A., and Witzel, K.-P. (1998). Development of PCR Primer Systems for Amplification of Nitrite Reductase Genes (nirK and nirS) To Detect Denitrifying Bacteria in Environmental Samples. *Appl. Environ. Microb.* 64, 3769.

Brosius, J., Dull, T., Sleeter, D., and Noller, H. (1981). Gene organization and primary structure of a ribosomal RNA operon from Escherichia coli. *J Mol Biol.* 148, 107–27.

Ettwig, K. F., Van Alen, K. ., Van de Pas-Schoonen, K. T., Jetten, M. S. M., and Strous, M. (2009). Enrichment and molecular detection of denitrifying methanotrophic bacteri of the NC10 phylum. *Appl. Environ. Microbiol.* 75, 3656–62.

Graham, D. W., Knapp, C. W., Van Vleck, E. S., Bloor, K., Lane, T. B., and Graham, C. E. (2007). Experimental demonstration of chaotic instability in biological nitrification. *The ISME journal* 1, 385–93.

Holmes, A. J., Costello, A., Lidstrom, M. E., and Murrell, J. C. (1995). Evidence that particulate methane monooxygenase and ammonia monooxygenase may be evolutionarily related. *FEMS Microbiol. Lett.* 132, 203–208.

Juretschko, S., Timmermann, G., Schmid, M., Schleifer, K. H., Pommerening-Röser, A., Koops, H., and Wagner, M. (1998). Combined molecular and conventional analyses of nitrifying bacterium diversity in activated sludge: Nitrosococcus mobilis and Nitrospira-like bacteria as dominant populations. *Appl. Environ. Microb.* 64, 3042–51.

Luesken, F. a, Zhu, B., Van Alen, T. a, Butler, M. K., Diaz, M. R., Song, B., Op den Camp, H. J. M., Jetten, M. S. M., and Ettwig, K. F. (2011). pmoA Primers for detection of anaerobic methanotrophs. *Appl. Environ. Microb.* 77, 3877–80.

Maixner, F., Nogurea, D. R., Anneser, B., Stoecker, K., Wegl, G., Wagner, M., and Daims, H. (2006). Nitrite concentration influences the population structure on Nitrospira-like bacteria. *Environ. Microbiol.* 8, 8:1487–1495.

McTavish, H., Fuchs, J. a, and Hooper, a B. (1993). Sequence of the gene coding for ammonia monooxygenase in Nitrosomonas europaea. *J. Bacteriol.* 175, 2436–44.

Neef, A., Amann, R., Schlesner, H., and Schleifer, K. H. (1998). Monitoring a widespread bacterial group: in situ detection of planctomycetes with 16S rRNA-targeted probes. *Microbiology* 144, 3257–3266.

Rotthauwe, J. H., Witzel, K. P., and Liesack, W. (1997). The ammonia monooxygenase structural gene amoA as a functional marker: molecular fine-scale analysis of natural ammonia-oxidizing populations. *Appl. Environ. Microb.* 63, 4704–12.

Schmid, M., Twachtmann, U., Klein, M., Strous, M., Juretschko, S., Jetten, M., Metzger, J. W., Schleifer, K. H., and Wagner, M. (2000). Molecular evidence for genus level diversity of bacteria capable of catalyzing anaerobic ammonium oxidation. *Syst. Appl. Microbiol.* 23, 93–106.

Schmid, M., Walsh, K., Webb, R., Rijpstra, W. I. C., Van de Pas-schoonen, K. T., Verbruggen, M. J., Hill, T., Moffett, B., Fuerst, J., Schouten, S., et al. (2003). Candidatus "Scalindua brodae", sp. nov., Candidatus " Scalindua wagneri ", sp . nov ., Two New Species of Anaerobic Ammonium Oxidizing Bacteria. *Syst. Appl. Microbiol.* 26, 529–538.

Throbäck, I. N., Enwall, K., Jarvis, Å., and Hallin, S. (2004). Reassessing PCR primers targeting nirS, nirK and nosZ genes for community surveys of denitrifying bacteria with DGGE. *FEMS Microbiology Ecology* 49, 401–417.

Wertz, S., Poly, F., Le Roux, X., and Degrange, V. (2008). Development and application of a PCR-denaturing gradient gel electrophoresis tool to study the diversity of Nitrobacter-like nxrA sequences in soil. *FEMS microbiology ecology* 63, 261–71.
